# Evaluation of the Long-Term Administration of Proton Pump Inhibitors (PPIs) in the Mineral Nutrient’s Bioavailability

**DOI:** 10.1021/acsomega.5c07700

**Published:** 2025-11-12

**Authors:** Andréa Santana de Brito, Angerson Nogueira do Nascimento, Fernando Luiz Affonso Fonseca, Alexandre Minami Fioroto, Giuliana Petri, Rafaela Garcia Vidigal do Nascimento

**Affiliations:** † Institute of Environmental, Chemical and Pharmaceutical Sciences (ICAQF), Federal University of São Paulo, CEP: 09972-270 São Paulo, Brazil; ‡ ABC Medical School, 125191 Faculty of Medicine of ABC—University Center (FMABC), CEP: 09060-870 Santo André, Brazil

## Abstract

Nutrient absorption in the human body can be influenced by factors, such as diet and medication use. The interaction between drugs and nutrients may lead to adverse effects, primarily due to reduced levels of essential elements. Therefore, evaluation of these interactions is important to prevent health complications. Proton pump inhibitors (PPIs), widely used to reduce gastric acid production, have been associated with interactions that may cause disorders. This study aims to assess the continuous use of PPIs and their effect on the bioavailability of Fe, Ca, Zn, Mg, Cu, and K in the diet. Rats were used as an animal model and divided into control and omeprazole-treated groups with subgroups based on treatment durations (10, 30, and 60 days). After each period, animals were euthanized and blood and organs were collected for analysis. Physiological, biochemical, and hematological parameters were evaluated. Elemental quantification was performed by using Inductively Coupled Plasma Mass Spectrometry (ICP-MS). The data revealed variations in hematological markers including reductions in red blood cells, hemoglobin, and hematocrit. Changes in the hematimetric indices and leukocyte counts were also observed. Elemental analysis showed imbalances in Fe, Cu, and Ca levels in both the blood and organs. These findings suggest that prolonged PPI administration may negatively affect nutrient availability and physiological stability, highlighting the importance of further investigation into the nutritional consequences of long-term PPI use resulting in conditions such as microcytic anemia, bone malabsorption, and other issues related to mineral deficiency.

## Graphical abstract

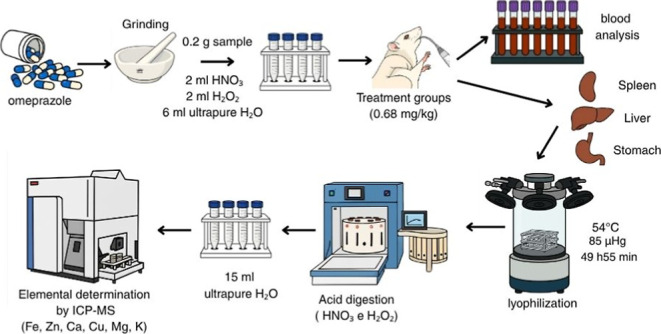

## Introduction

The human body requires a diverse array of nutrients, each fulfilling distinct physiological roles that underpin both health and disease prevention. These compounds, broadly divided into macronutrients such as proteins, carbohydrates, fats, and micronutrients, are obtained primarily through the diet. While macronutrients are consumed in larger quantities to supply energy and support tissue growth and repair, micronutrients, including a variety of vitamins and minerals, are needed in much smaller amounts.^1–4^

Minerals can be further subdivided into macroelements and microelements, depending on the amounts required by the body.^5^ Macroelements such as calcium, magnesium, and potassium are needed in higher concentrations and participate in structural, neuromuscular, and hydroelectrolytic balance functions. Microelements, including iron, zinc, and copper are required in trace amounts, however, they play crucial roles as enzyme cofactors, antioxidants, and regulators of immune function.^6^

Among these minerals, calcium is critical for blood clotting, neuromuscular excitability, and nerve impulse transmission. Iron is essential for hemoglobin production and is a component of enzymes such as cytochrome oxidase, catalases, and dehydrogenases found in skeletal muscle.^7–9^ Copper contributes to iron mobilization for hemoglobin synthesis and functions as a catalytic cofactor of cuproenzymes, which are necessary for cellular respiration, neurotransmitter biosynthesis, antioxidant defense, and connective tissue formation.^10,11^ Zinc is involved in cell replication, phagocytic activity, sexual maturation, fertility, and reproduction.^12^ Magnesium and potassium are essential for cardiac function, skeletal muscle contraction, and cellular respiration.^13–15^ Deficiency in these nutrients can lead to disorders such as anemia, osteoporosis, arrhythmia, and chronic kidney disease.^16–18^

Considering the importance of these elements in the human diet and their contributions to physiological functions, numerous bioaccessibility studies have been conducted to identify and quantify nutrients in foods.^19^ However, while total nutrient concentrations provide insights into the chemical composition of foods, they do not necessarily reflect the amounts absorbed by the body.^1,2^ Several factors influence nutrient bioavailability, one of which is the use of medications. For instance, proton pump inhibitors (PPIs) are particularly relevant in this context, as they are widely prescribed and concerns have been raised regarding their potential overuse.^20–23^ Omeprazole, one of the most prescribed PPIs, is recommended for short-term use, typically not exceeding 8 weeks. Nevertheless, chronic and unregulated consumption is frequently observed, raising concerns about possible health risks.^24^

PPIs act by increasing intragastric pH and significantly reducing hydrogen ion concentration, thereby hindering nutrient bioavailability during gastric transit.^25,26^ Their use has been associated with adverse health impacts, including increased risk of infections due to suppressed gastric acidity, alterations in gastric microbiota, and bacterial overgrowth in the small intestine.^27,28^ Moreover, long-term omeprazole administration may negatively affect kidney function, although the immunological mechanisms underlying PPI-induced toxicity remain unclear.^29,30^

Current evidence suggests a potential impact of PPIs on nutritional status, which is potentially associated with undernutrition. However, existing studies in this field remain inconsistent. For instance, a study conducted on older hospitalized patients demonstrated no significant association between long-term PPI use and undernutrition. Conversely, in certain studies, PPI administration has been linked to weight gain. Additionally, a cardiology study indicated an association between PPIs and increased nutritional risks among patients undergoing rehabilitation following treatment for ischemic and valvular heart disease. These differences highlight the need for further investigations to evaluate the effects of PPIs on nutritional status.^31,32^

In this context, this study aims to investigate the relationship between omeprazole administration and its potential effects on the absorption and bioavailability of Fe, Ca, Mg, Zn, Cu, and K. Furthermore, physiological parameters and hematological profiles were assessed in rats to provide complementary insights.

## Materials and Methods

### Drug and Animal Model

All experimental procedures were conducted in accordance with the Ethical Principles in Animal Experimentation, as established by the National Council for the Control of Animal Experimentation (CONCEA). The protocol was reviewed and approved by the Ethics Committee on Animal Use of the Faculty of Medicine of ABC (CEUA—FMABC).

Thirty-six adult male Wistar rats (200–300 g) were used. The animals were randomly assigned to six groups (*n* = 6): (1) Control-10 days, (2) Control-30 days, (3) Control-60 days, (4) Treatment-10 days, (5) Treatment-30 days, and (6) Treatment-60 days. Each group was housed in individually labeled cages.

The PPI used in this study was omeprazole (Geolab, Anápolis, GO, Brazil). Animals in the treatment group received a daily oral gavage of omeprazole at a dose of 0.68 mg/kg, with ad libitum access to water and Nuvital CR-1 feed. The drug solution was prepared according to Larsson et al.^26^ Briefly, omeprazole granules were ground with a mortar and dispersed in a vehicle containing 0.25% hydroxyethylcellulose 4400 in 0.1 M sodium bicarbonate (pH ≈ 7.4). Control groups received the vehicle solution.

At the end of each treatment period (10, 30, or 60 days), the animals were euthanized with sodium thiopental (100 mg/kg, intraperitoneally). Blood was collected by caudal vena cava puncture and transferred to tubes containing separating gel and EDTA. Samples were centrifuged at 3500 rpm for 10 min, and complete blood counts were performed immediately. Following euthanasia, the liver, spleen, and stomach were dissected, fragmented, and stored in Eppendorf tubes properly labeled with animal number, treatment duration, and organ identify.

### Biochemical and Hematological Analyses

Peripheral blood cell analysis (hemogram) was performed using an ABX PENTRA 120 Horiba analyzer, which applies flow cytometry to quantify erythrocytes, leukocytes, and platelets.

Biochemical parameters were measured with the Cobas 6000 analyzer series (Roche Diagnostics), which uses a colorimetric method with fully automated spectrophotometric detection, enabling both biochemical and immunological analyses.

### Sample Preparation

Samples were lyophilized using a benchtop freeze-dryer (model L108, Liotop, São Carlos, Brazil) equipped with a vacuum pump. Drying was carried out at −54 °C under constant pressure for approximately 48 h.

Lyophilized samples were digested with a closed-vessel microwave digestion system (Milestone, Sorisole, Italy). Approximately 0.1 g of spleen and 0.2 g of stomach and liver were weighed, considering tissue density and composition. Higher sample masses were required for the liver and stomach due to their greater density and structural complexity, ensuring efficient digestion. Perfluoralkoxy (PFA) vessels (100 mL) were used, with an acid mixture containing 2.0 mL of HNO_3_, 2.0 mL of H_2_O_2_, and 6.0 mL of ultrapure H_2_O.

The digestion program was conducted in four steps: first (80 °C, 5 min, 2 min hold), second (140 °C, 5 min, 2 min hold), third (190 °C, 5 min, 10 min hold), and fourth (220 °C, 2 min, 29 min hold). Vessels were cooled for 30 min before opening to ensure safety. The resulting solutions had a pH of approximately 2, which was suitable for ICP–MS analysis.

### Elemental Determination in Biological Samples

The solutions resulting from acid digestion were analyzed by inductively coupled plasma mass spectrometry (ICP–MS, iCAP Q, Thermo Fisher Scientific, Cambridge, England) equipped with a quadrupole mass analyzer. Calibration and internal standard solutions for ICP–MS analysis were prepared from multielemental solutions (G1516 V and MICPG1583V, Quimlab Produtos de Química Fina Ltda, São José dos Campos, Brazil). Calibration and internal standard solutions were obtained by serial dilutions within the concentration range of 0.1–100 ppb. The internal standard concentration was fixed at 50 ppb. Intermediate solutions were prepared in 0.1% HNO_3_, with deionized water used for dilution. Linear regression was applied, and the limits of detection (LOD) and quantification (LQ) were calculated from ten measurements of the analytical blank. The instrumental parameters used in the operation of ICP–MS are described in Table 1.

**Table 1:** Instrumental Parameters Used in ICP–MS

parameter	operational condition	unit
radio frequency generator	27	MHz
radio frequency power	1.5	kW
plasma gas flow	1.8	L min^–1^
auxiliary gas flow	1.8	L min^–1^
nebulizer gas flow	1.1	L min^–1^
sampling depth	7	mm
integration time	3	s
nebulizer	concentric	
spray chamber	cyclonic	
number of replicates	3	
analyzed isotopes	^24^Mg,^39^K,^44^Ca,^57^Fe,^65^Cu,^66^Zn	

### Statistical Analysis

Experimental data were analyzed using two-way analysis of variance (ANOVA). Post hoc comparisons were performed with Tukey’s test. Significance was considered when *p* <0.05. Additionally, effect sizes were calculated using Cohen’s d and classified as trivial (*d* <0.2), small (0.2 ≤ *d* < 0.5), moderate (0.5 ≤ *d* < 0.8), or large (*d* ≥ 0.8).

## Results and Discussion

Hematological and biochemical parameters were analyzed by comparing treated and control groups of Wistar rats, selected due to its physiological and genetic similarities to humans, which facilitate the evaluation of drug-related effects, including changes in blood cell profiles and mineral levels.^33,34^ Table 2 summarizes the hematological findings for each experimental group.

**Table 2:** Hematological Parameters Evaluated during 60 Days of Treatment with Omeprazole^a^

	treatment time											
	10 days				30 days				60 days			
	control (*n* = 6)		treated (*n* = 6)		control (*n* = 6)		treated (*n* = 6)		control (*n* = 6)		treated (*n* = 6)	
parameters	mean	±SD	mean	±SD	mean	±SD	mean	±SD	mean	±SD	mean	±SD
WBC (10^3^ μL)	5.65	2.41	6.42	1.19	5.80	1.76	6.05	0.61	4.31	0.36	5.47	0.89
RBC (10^6^/μL)	8.61	1.01	9.40	0.73	9.09	0.80	9.16	0.55	9.23	0.39	9.08	0.35
HGB (g/dL)	15.97	0.72	16.38	0.92	15.63	0.98	15.62	1.10	15.47	0.33	15.17	0.45
HCT (%)	51.08	3.11	52.17	3.60	50.02	3.96	50.97	5.05	49.43	1.34	48.22	0.79
MCV (fL)	56.24	2.91	55.55	1.58	55.08	1.71	55.55	2.50	53.58	1.33	53.17	1.89
MCH (pg)	17.53	0.43	17.45	0.60	17.25	0.65	17.03	0.39	16.77	0.41	16.72	0.33
MCHC (g/dL)	31.28	0.62	31.45	0.64	31.30	0.64	30.70	0.88	31.28	0.41	31.47	0.79
PLT (10^3^/μL)	719.0	152.2	790.2	74.0	727.7	70.4	723.5	120.4	614.8	99.9	686.3	143.3
RDW-SD (fL)	22.90	1.84	22.00	1.03	23.88	1.47	23.78	2.09	23.72	0.98	25.63	5.05
RDW-CV (%)	15.62	1.82	16.07	2.01	16.98	1.47	17.08	0.88	17.88	0.56	18.72	1.73
MPV (fL)	8.15	0.36	7.68	0.24	7.47	0.22	7.77	0.38	7.90	0.41	7.73	0.24
NRBC (10^3^/μL)	0.01	0.01	0.02	0.01	0.02	0.00	0.01	0.01	0.01	0.00	0.02	0.01
NEUT (10^3^/μL)	0.52	0.23	0.57	0.14	0.41	0.11	0.69	0.27	0.46	0.08	0.89	0.46
LYMPH (10^3^/μL)	4.97	2.25	5.67	1.16	4.98	1.25	5.17	0.53	3.69	0.38	4.44	0.90
MONO (10^3^/μL)	0.09	0.10	0.07	0.03	0.36	0.56	0.09	0.06	0.03	0.01	0.05	0.06
EO (10^3^/μL)	0.08	0.03	0.09	0.02	0.05	0.03	0.09	0.03	0.12	0.14	0.09	0.05
BASO (10^3^/μL)	0.01	0.01	0.02	0.01	0.01	0.01	0.01	0.01	0.01	0.01	0.00	0.01
Ca (mg/dL)	10.52	0.63	11.28	0.71	9.42	0.47	10.04	0.84	8.85	0.31	8.76	0.52
Fe (μg/dL)	209.6	42.7	189.1	8.55	211.5	54.13	195.6	54.78	215.1	22.72	180.23	26.22
Mg (mg/dL)	3.33	0.53	3.86	0.45	2.54	0.42	2.96	0.89	2.65	0.42	2.54	0.77
K (mmol/L)	4.88	0.35	5.30	0.96	5.01	1.07	4.80	0.84	4.63	1.39	4.39	1.08

a **WBC:** White blood cell; **RBC:** Red blood cell; **HGB:** Hemoglobin; **HCT:** hematocrit; **MCV:** Mean corpuscular volume, **MCH:** mean corpuscular hemoglobin, **MCHC:** mean corpuscular hemoglobin concentration **PLT:** platelets; **RDW-SD:** Red Cell Distribution Width–standard deviation; **RDW-CV:** Red Cell Distribution Width–coefficient of variation; **MPV:** mean platelet volume; **NRBC:** nucleated red blood cell; **NEUT:** neutrophil; **LYMPH:** lymphocyte; **MONO:** monocyte; **EO:** eosinophil; **BASO:** basophil.

These parameters constitute a complete blood count, serving as a diagnostic tool for various diseases such as iron deficiency anemia, allergies, and infections.^35^ They provide both quantitative and qualitative information about blood components, including red blood cells (RBCs), white blood cells (WBCs, or total leukocyte count), and hematimetric indices. These parameters are essential for diagnosing anemia and evaluating risks of bleeding or infections.^36,37^

Significant alterations were observed in RBC and WBC counts as well as in hematimetric indices, hemoglobin levels, and hematocrit values. The following sections present these results in detail.

### Anemia Evaluation

The evaluation of anemia was conducted through analysis of red blood cell (RBC) parameters, including hemoglobin (HBG), hematocrit (HCT), red cell distribution width (RDW), and iron (Fe) concentration (Figure 1). These parameters are essential for diagnosing anemia, differentiating between its forms, and assessing bleeding risk.^36,37^

**Figure 1:**
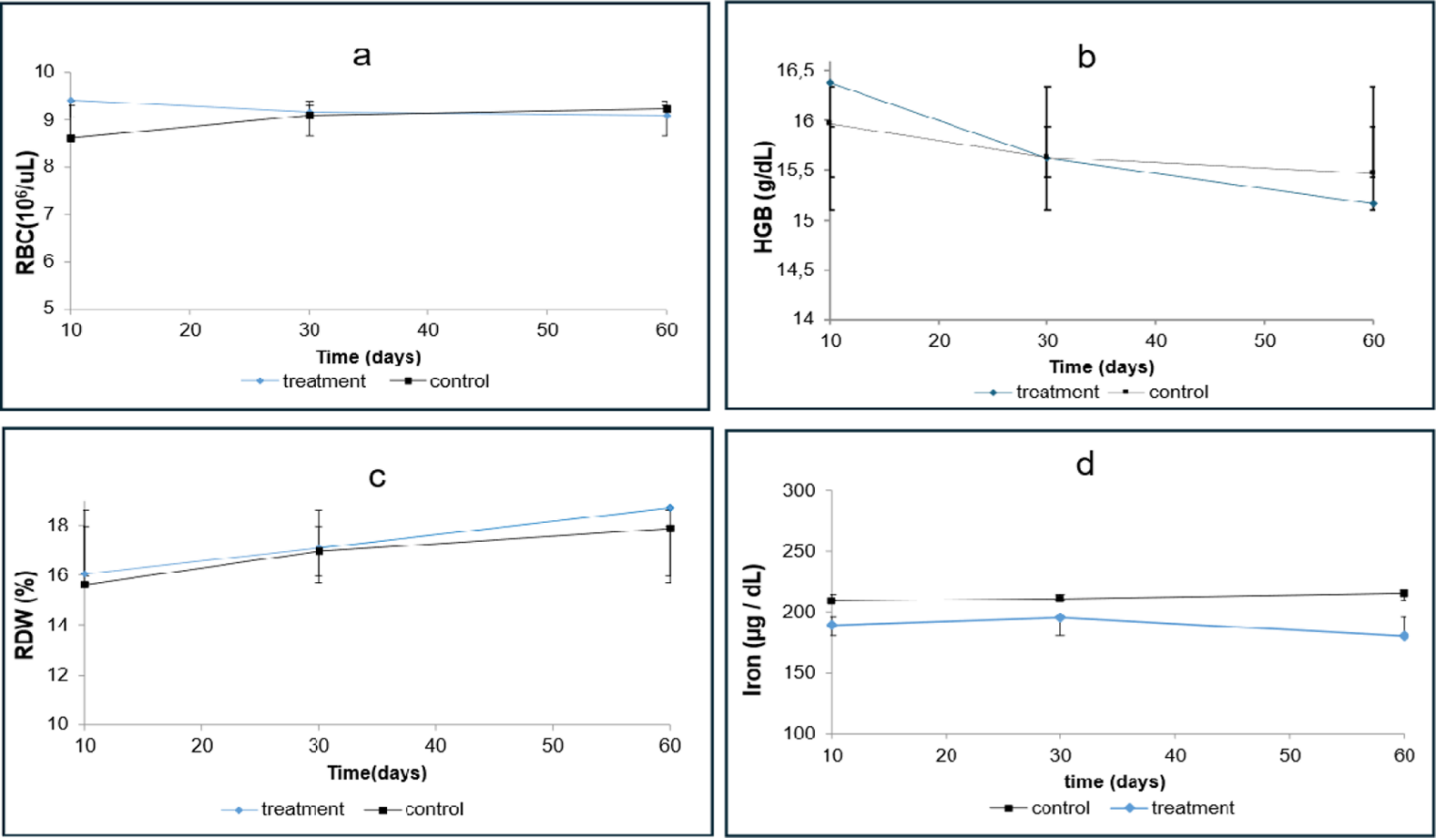
Effect of the omeprazole on: (a) Red Blood Cell concentrations (10^6^/μL); (b) Hemoglobin concentrations (g/dL); (c) Red Cell Distribution Width (%); and (d) Iron concentrations (μg/dL). Values obtained from Two-way ANOVA (*p* <0.05).

The RBC count (Figure 1a) showed a gradual reduction over the 60 day treatment period. After 10 days of omeprazole administration, the mean concentration was 9.40 × 10^6^/ μL, decreasing to 9.16 × 10^6^/ μL and 9.08 × 10^6^/ μL at 30 and 60 days, respectively. Although the recommended duration of omeprazole therapy in humans is limited to a maximum of 60 days, prolonged and indiscriminate use is frequently reported.^24^ To confirm anemia in the treated groups, additional HGB (Figure 1b) and RDW (Figure 1c) assessments were performed.

HGB values decreased progressively during omeprazole treatment, from 16.38 g/dL on day 10 to 15.62 g/dL on day 30 and 15.17 g/dL on day 60 (Figure 1b). These findings suggest that prolonged drug use may reduce HGB levels, supporting its use as an indicator for anemia. It should be noted, however, that reference thresholds for anemia in experimental rat models are not standardized. For humans, anemia is clinically defined as hemoglobin levels below 13.0 g/dL in men and below 12.0 g/dL in nonpregnant women.^38^ In this study, anemia was assessed through a relative comparison with the control group rather than absolute thresholds.

Two-way analysis of variance (ANOVA) indicated significant differences between groups, with omeprazole demonstrating a moderate effect on HGB values after 60 days (0.5 ≤ *d* < 0.8). These findings suggest that continuous use of the drug can lead to long-term reductions in HBG concentration, potentially causing anemia. However, evaluations in RDW (Figure 1c) and Fe levels (Figure 1d) are required to identify specific types of anemia.

Regarding Fe levels (Figure 1d), the treated group exhibited lower concentrations after 60 days of treatment compared with controls (180.23 μg/dL vs 215 μg/dL). Two-way ANOVA, followed by Tukey’s test confirmed significant differences among groups. Furthermore, effect size analysis indicated a large impact (Cohen’s d) in the long term, suggesting that omeprazole may negatively influence Fe concentrations as early as day 10 of the experiment.

RDW values (Figure 1c) increased progressively during treatment, from 16.07% at day 10 to 17.08% on day 30 and 18.72% at day 60, with consistently higher levels than controls (15.62%, 16.98%, and 17.88%, respectively). The observed increase indicates enhanced heterogeneity of RBC size. When evaluated together with indices such as mean corpuscular volume (MCV), RDW provides additional information for differentiating anemia types, such as microcytic or macrocytic anemia.^39^

Finally, significant interference in iron homeostasis was observed, consequently affecting erythropoiesis, as evidenced by the decrease in HGB and increase in RDW levels. These mechanisms should be carefully considered when addressing the effects associated with the prolonged use of PPIs.

### Inflammatory Response

The evaluation of white blood cells (WBCs) included both total leukocyte counts and differential subtypes such as lymphocytes, neutrophils, and eosinophils. Each of these subpopulations plays a distinct role in immune function. Lymphocytes are central to adaptive immunity, contributing to antibody production (B lymphocytes) and immune regulation (T lymphocytes).^40^ Neutrophils represent the first line of defense against infectious agents, particularly bacteria, through phagocytosis. Eosinophils, in turn, participate in responses to parasites and allergic reactions by releasing inflammatory mediators from their granules.^41^ The combined analysis of these parameters enables the identification of changes in immunological and inflammatory profiles and is therefore useful for evaluating the effects of drug exposure.^42,43^

In the present study, a significant increase in total WBCs was observed in the group treated with omeprazole, with values of 6.42 × 10^3^/μL at 10 days, 6.05 × 10^3^/μL at 30 days, and 5.47 × 10^3^/μL at 60 days. By contrast, the control group showed values of 5.65 × 10^3^/μL, 5.80 × 10^3^/μL, and 4.31 × 10^3^/μL at the same time points. Alterations were also noted in lymphocyte concentrations, with decreased progressive in the treated animals: 5.67 × 10^3^/μL (10 days), 5.17 × 10^3^/μL (30 days), and 4.44 × 10^3^/μL (60 days). These findings, illustrated in Figure 2 (a and b), suggest a potential immunological response associated with omeprazole administration.

**Figure 2:**
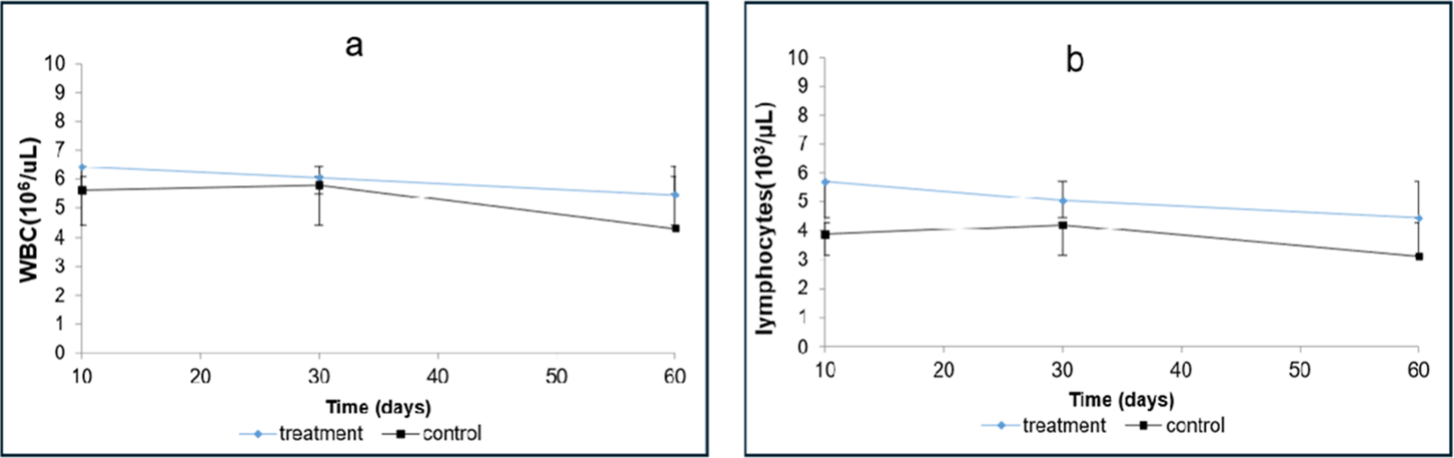
Effect of the omeprazole on: (a) White Blood Cell concentrations (g/dL) and (b) Lymphocyte concentrations. Values obtained from Two-way ANOVA (*p* <0.05).

These alterations may reflect disruptions in the immune system in the treated rats. Previous studies have reported that omeprazole can destabilize the immune system, potentially compromising the bactericidal activity of the defense cells through mechanisms that remain unclear. This effect may represent a particular risk for individuals with pre-existing immunosuppression. Furthermore, it has been proposed that omeprazole-induced changes may promote bacterial proliferation, leading to adverse clinical outcomes.^44^

Additional evidence links omeprazole use to an increased risk of bacterial pneumonia. This may be explained by gastric pH elevation, which facilitates bacterial migration.^45,46^ Under normal physiological conditions, gastric acid contributes to host defense by inactivating microorganisms ingested with food. By markedly reducing gastric acid secretion, omeprazole may create a more favorable environment for microbial survival and increase risk of gastrointestinal infections.^45,47^

Prolonged omeprazole use has also been associated with a higher incidence of community-acquired pneumonia, possibly due to the aspiration of gastric contents. Moreover, suppression of gastric acidity may facilitate colonization of the respiratory tract by pathogenic bacteria.^44,46^ An increase in bacterial growth typically triggers leukocytosis, as new lymphocyte populations are generated in response to viral or bacterial antigens.^36,37^ In this context, the disparities observed between the omeprazole-treated and control groups suggest the potential induction of an inflammatory response by the drug. This observation is particularly relevant, given the central role of lymphocytes in modulating inflammation.

Nonetheless, the complex relationship between omeprazole and immune regulation requires further investigation.^47^ These findings should therefore be evaluated with caution. Omeprazole should not be regarded as a therapeutic strategy to enhance immune responses. Instead, the data highlight important avenues for future research and may have potential clinical implications.

### Elemental Determination in Biological Samples

The distribution of Fe, Cu, Mg, Zn, Ca, and K in different tissues was investigated to evaluate the effects of omeprazole administration on elemental homeostasis in rats. Synchrony among nutrient absorption, usage, and storage is essential for maintaining the physiological balance. Hence, a comprehensive evaluation of these elements is critical for identifying potential disturbances caused by drug exposure.

Liver, stomach, and spleen samples were analyzed using ICP–MS. Despite the limited literature available on the effects of drugs on minerals and trace element concentrations in humans and animals, the application of ICP–MS for elemental determination in these tissues holds significant clinical and scientific relevance.^48–50^ These organs were selected due to their essential roles in physiological processes. Elemental analysis provides valuable insights into their chemical composition, particularly given their participation in blood formation. Substantial alterations in their elemental composition may result in physiological complications.^9,10,51^ The results are presented in Figures 3–5, with detailed discussions for each organ provided in the following sections.

**Figure 3:**
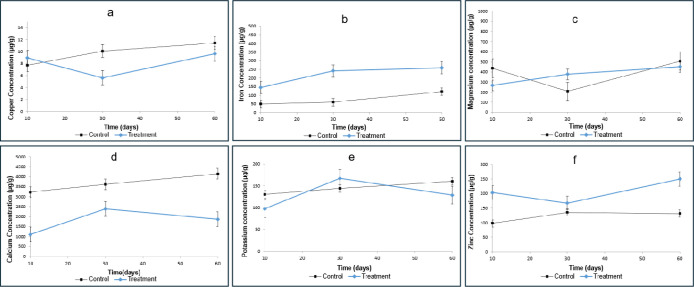
Mean concentrations of Cu, Mg, K, Zn, Fe, and Ca in liver determined by ICP–MS. Values are presented as mean ± standard deviation (μg/g). (a) Copper; (b) iron; (c) magnesium; (d) calcium; (e) potassium; and (f) zinc. The control group is represented in black, and the treatment group in blue. Statistical significance was determined by one-way ANOVA (*p* <0.05).

### Liver Mineral Concentration

The liver plays a crucial role in the human body, contributing to metabolic regulation, immune modulation, digestion, detoxification, and vitamin storage.^52^ Although it is not directly responsible for hematopoiesis, as occurs in the bone marrow, the liver exerts a significant influence on mineral metabolism and on the synthesis of substances essential for blood cell formation. This indirect regulation may affect the occurrence of anemia and other diseases.^53^

Lower hepatic copper concentrations were observed in the omeprazole-treated group compared to the control group, particularly after 30 days of treatment (5.62 μg·g^–1^ vs 10.09 μg·g^–1^, respectively) as illustrated in Figure 3a. These findings suggest an imbalance in copper homeostasis and a reduction in hepatic copper fixation. Copper reduction may affect intestinal iron absorption, reducing its bioavailability and contributing to anemia, as evidenced by hematological analysis of rats and reduced iron levels in the blood.^10,11,54^ This outcome correlates with anemia, as evidenced by reductions in hemoglobin levels and red cell counts in the hematological analysis of rats (Figure 1a,b), along with the previously discussed decrease in blood Fe levels (Figure 1d).

Additionally, hepatic iron concentrations were higher in the treated group, reaching 258.92 μg·g^–1^ after 60 days of omeprazole exposure compared with 60.04 μg·g^–1^ in the control group (Figure 3b). Smaller increases were also noted on days 10 and 30 days. These results may reflect compensatory hepatic accumulation in response to reduced iron levels. Some studies suggest that prolonged hepatic iron accumulation has been associated with tissue injury and cirrhosis, which can disrupt the synthesis of proteins necessary for red blood cell formation and contribute to anemia.^55,56^

Magnesium concentrations in the liver also showed a progressive increase during treatment, with hepatic levels rising from 265.98 μg·g^–1^ at the baseline to 449.1 μg·g^–1^ after 60 days (Figure 3c). The liver functions as a temporary reservoir for magnesium, with bones representing the primary storage site. Elevated hepatic magnesium may therefore indicate altered distribution associated with long-term drug exposure. Omeprazole use has frequently been linked to systemic magnesium deficiency, which may in turn disrupt calcium balance.^57–59^ This occurs through reduced secretion and activity of parathyroid hormone (PTH), the main regulator of calcium homeostasis.^60,61^ Accordingly, calcium variations were also examined.

Calcium concentrations in the liver were lower in omeprazole-treated rats compared to those of controls at all time points (Figure 3d). After 60 days, hepatic calcium reached 1866.16 μg·g^–1^ in the treated group, compared with 4150.79 μg·g^–1^ in the controls. Approximately 99% of total body calcium is stored in bones and teeth, with only 1% circulating in the blood and tissues such as the liver. These results suggest a redistribution mechanism. Indeed, plasma calcium levels (Table 2) were increased in the treated group, reaching 10.04 mg/dL after 30 days, compared to 9.42 mg/dL in controls, possibly indicating bone resorption induced by omeprazole, as reported in the literature.^17,20,57^ The reduction in hepatic calcium may be related to the mobilization of calcium from bone tissue to maintain blood homeostasis. This redistribution, possibly stimulated by omeprazole, suggests decreased hepatic retention and systemic regulatory changes in calcium metabolism, as discussed by other authors.^58^

Potassium levels in the liver (Figure 3e) displayed a different pattern. Control animals exhibited a constant increase over time (130.61 μg·g^–1^ to 160.16 μg·g^–1^ at 60 days), whereas treated animals showed an increase at 30 days (167.53 μg·g^–1^), followed by a decrease at 60 days (128.86 μg·g^–1^). Potassium is an essential mineral present in all cells, including liver cells (hepatocytes).^15^ However, the specific potassium concentration in the liver and its function in this organ are not fully understood. The liver plays an important role in regulating potassium balance in the body, encompassing both absorption and excretion.^59^ Although the direct relationship between omeprazole use and liver potassium concentration is not extensively studied or documented, some studies have examined the effects of omeprazole on mineral absorption, such as Mg, which participates in potassium transport in body tissues.^60–62^

These studies concluded that omeprazole, as a proton pump inhibitor, can reduce gastric acidity, which can impact magnesium absorption in the intestine. The resulting hypomagnesemia can lead to impaired potassium regulation, as magnesium is necessary for the effective transport of potassium into cells.^63^

Furthermore, by comparing the variation in potassium concentration with the Mg variation (Figure 3c), similarities between the two graphs can be inferred, with lower concentration in 10 days and 60 days of treatment compared to the control group, being able to demonstrate the influence of omeprazole on the absorption of the two minerals in the long term.

Hepatic zinc concentrations were higher in the tested group compared with controls across all time points (250.08 μg·g^–1^ vs 131.57 μg·g^–1^, Figure 3f). Unlike other minerals, zinc is not stored in a specific organ but is distributed to tissues according to functional demand, especially during inflammatory or infectious processes.^12,54^ The observed increase may reflect altered zinc homeostasis induced by omeprazole, resulting in enhanced uptake or retention in the liver. Although direct evidence is limited, gastrointestinal drugs are known to affect micronutrient bioavailability.^64^ Zinc also serves as a cofactor for antioxidant enzymes, such as superoxide dismutase (SOD), which protects against reactive oxygen species. Elevated hepatic zinc may therefore represent a compensatory mechanism to limit oxidative damage.^65^ Indeed, inflammatory conditions and oxidative stress are known to increase zinc demand in specific tissues, including the liver.^66^

### Stomach Mineral Concentration

Although the stomach does not directly store minerals, it plays an indirect role in the development of diseases related to the poor absorption of dietary minerals. Therefore, the elemental analysis of this organ facilitates the identification of potential changes in nutrient absorption.^59^ Figure 4 illustrates the concentrations of the evaluated elements in the stomach of the experimental animals.

**Figure 4:**
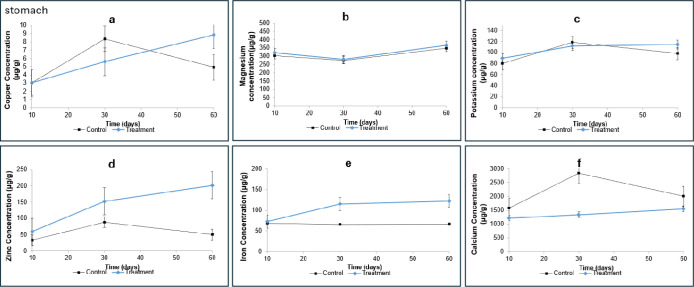
Mean concentrations of Cu, Mg, K, Zn, Fe, and Ca in stomach determined by ICP–MS. Values are presented as mean ± standard deviation (μg/g). (a) Copper; (b) magnesium; (c) potassium; (d) zinc; (e) iron; and (f) calcium. The control group is represented in black, and the treatment group in blue. Statistical significance was determined by one-way ANOVA (*p* <0.05).

The average concentration of copper in the rat’s stomach (Figure 4a) was higher in omeprazole-treated animals, particularly after 60 days of administration (8.87 μg·g^–1^ versus 4.91 μg·g^–1^ in the control group), suggesting copper accumulation with prolonged treatment. An *in vivo* study assessing the effects of reducing gastric pH on copper metabolism using antacids reported a similar increase in gastric copper levels compared to controls, indicating interference in copper solubilization and absorption.^64^

Copper is primarily absorbed in the small intestine, but gastric acidity is essential for converting dietary copper into soluble forms (Cu^2^), which facilitates intestinal absorption.^58^ When gastric acid production is inhibited by omeprazole, copper solubilization decreases, leading to reduced absorption and increased gastric retention.^67^

The body maintains a strict balance of copper levels, regulating both absorption and excretion. Changing the gastric pH can interfere with this balance, resulting in an anomalous distribution of copper in tissues. The presence of elevated copper in the stomach may be a reflection of this homeostatic dysregulation, where the body cannot absorb copper efficiently, leading to local accumulation.^68^

Magnesium concentrations in stomach (Figure 4b) showed no significant differences between control and treated groups across all time points (e.g., 367.24 μg·g^–1^ at 60 days in treated animals versus 349.58 μg·g^–1^ in controls). These findings indicate that omeprazole had no major impact on gastric magnesium levels, consistent with previous reports.^64^ Magnesium is mainly absorbed in the small intestine, and its uptake is less dependent on gastric acidity than that of iron or calcium, which may explain the absence of significant changes in this study.^58,63^

Similar results were observed for potassium concentration in the rat stomach (80.89 μg·g^–1^ in the group treated for 10 days versus 89.74 μg·g^–1^ in controls; Figure 4c), indicating minimal influence of omeprazole on gastric K levels despite its role in ionic exchanges mediated by the H^+^/K^+^-ATPase proton pump.^69^ The gastric proton pump mediates the exchange of hydrogen and potassium ions to enable hydrochloric acid (HCl) secretion. Although omeprazole inhibits this pump and effectively suppresses acid production, gastric potassium concentrations are not substantially altered due to the potassium involved in the exchange is recycled rather than eliminated.^59^

The potassium required for pump activity is readily available and is recaptured by gastric cells for continuous use, minimizing the likelihood of significant fluctuations in tissue concentrations. Moreover, systematic potassium homeostasis is tightly regulated through intestinal absorption and renal excretion. Consequently, even with omeprazole-induced inhibition of acid secretion, potassium concentrations in the stomach and others tissues remain stable.^58,59,63^

Figure 4d shows zinc concentrations in the stomach, which were consistently higher in omeprazole-treated animals compared to controls, reaching 201.85 μg·g^–1^ versus 50.31 μg·g^–1^ after 60 days. This increase suggests zinc accumulation in the gastrointestinal tract, which appears to be intensified by prolonged drug exposure. Zinc levels in gastric cells are naturally high due to its role as a cofactor in several digestive enzymes.^65^ Previous studies have reported similar increased gastric zinc concentrations associated with reduced gastric pH following omeprazole administration.^64^

This finding may be explained by the biological role of zinc in enzymatic processes and physiological adaptations to altered gastric conditions. Zinc is an essential cofactor for enzymes such as metalloproteinases and peptidases, which are involved in protein breakdown and other digestive processes. These enzymes are abundant in gastric cells, and alterations in gastric acidity may enhance zinc demand to maintain adequate enzymatic activity. As a result, greater zinc uptake by gastric cells may occur under omeprazole treatment.^65,66,68,70^

The concentration of Fe in the stomach of the rats is shown in Figure 4e. The treated group exhibited Fe levels higher than those of the control group at all treatment periods (121.99 μg·g^–1^ versus 66.35 μg·g^–1^, at 60 days). One-way ANOVA revealed a statistically significant difference between the treated and control groups (*p* <0.05). This increase in Fe concentration may have been induced by omeprazole administration and could be further exacerbated by prolonged use of the drug. Iron is a crucial component of hemoglobin, responsible for oxygen transport in red blood cells, and the stomach contributes to iron absorption by converting nonheme iron into a more bioavailable form.^9,16,54^ The reduction in HCl secretion caused by omeprazole may impair this conversion, leading to Fe accumulation in stomach cells. Consistent with this mechanism, Naveh (1987) reported that prolonged use of antacids increases the iron concentration due to altered mineral metabolism.

The concentration of calcium in the stomach samples is shown in Figure 4f. Rats treated with omeprazole had lower Ca concentrations compared with the control group after 60 days of treatment (2005.33 μg·g^–1^ versus 1552.38 μg·g^–1^, respectively). One-way ANOVA indicated a statistically significant difference between groups (*p* <0.05). Considerable variation in calcium concentrations was observed throughout the treatment period. Previous studies on medications that reduce gastric acidity have reported higher gastric Ca concentration in treated groups, which contrasts with the present findings and requires further investigation.^64^

This discrepancy may be attributed to differences in calcium metabolism, the specific pharmacological action of omeprazole, and variations in the experimental conditions. Calcium absorption depends on solubility, which is maximized in an acidic environment. Under normal conditions, the acidic pH of the stomach helps to solubilize dietary calcium, facilitating its absorption in the intestine.^58^ When gastric acidity is reduced by omeprazole, calcium solubility decreases, which may result in impaired absorption and lower gastric Ca concentrations, as observed in this study.^61^

In contrast, studies reporting increased gastric calcium concentrations following pH reduction may have involved different experimental conditions such as the administration of different antacids or dietary variations in the animals studied. These factors influence the amount of calcium available in the gastrointestinal tract and its retention in the stomach.^64^ In this study, omeprazole specifically reduced gastric activity without direct calcium supplementation, which may explain the lower gastric calcium concentrations observed.

### Spleen Mineral Concentration

The spleen was the final organ subjected to ICP–MS analysis. A significant increase in the concentration of all minerals in the omeprazole-treated group was observed during all treatment periods, as shown in Figure 5, suggesting potential accumulation in the spleens of rats exposed to omeprazole. The spleen plays a crucial role in the immune system as a secondary lymphoid organ responsible for the production, storage, and activation of immune cells.^71^ Additionally, it serves as a temporary blood reservoir and filters damaged blood cells. Inflammation or injury to the spleen can result in a transient increase in mineral concentrations as part of the inflammatory or tissue repair response.^59^

**Figure 5:**
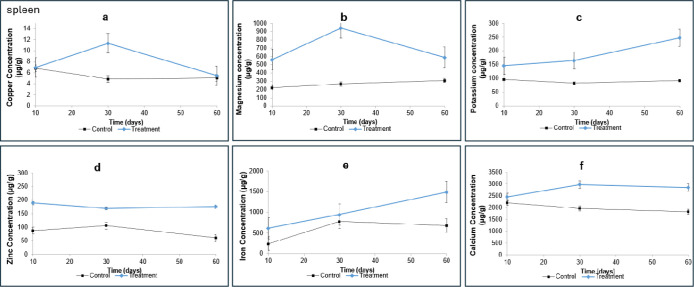
Mean concentrations of Cu, Mg, K, Zn, Fe, and Ca in spleen determined by ICP–MS. Values are presented as mean ± standard deviation (μg/g). (a) Copper; (b) magnesium; (c) potassium; (d) zinc; (e) iron; and (f) calcium. The control group is represented in black, and the treatment group in blue. Statistical significance was determined by one-way ANOVA (*p* <0.05).

Figure 5a shows the copper concentrations in the spleens of rats. A marked difference between the treated and control groups was observed after 30 days of treatment (11.33 μg·g^–1^ versus 4.84 μg·g^–1^, respectively), indicating a significant effect of omeprazole on copper accumulation. The spleen, which serves as a reservoir for Cu, typically contains elevated levels of this element. However, previous studies did not report significant differences in Cu concentration in rats treated with antacids.^64^

Omeprazole alters gastric acidity, which can impair intestinal copper absorption and bioavailability, leading to redistribution of the element among organs, including the spleen.^67,68^ The increased copper concentration in the spleen was observed after 30 days of omeprazole administration, indicating compensatory redistribution aimed at maintaining copper homeostasis in other tissues.^68^

Unlike copper, magnesium is not typically stored in the spleen, as its primary reservoirs are the bones and liver. Therefore, the increase in magnesium observed in the spleen of omeprazole-treated rats (943.08 μg·g^–1^ versus 266.72 μg·g^–1^ in controls, Figure 5b) is uncommon and not well-documented. Magnesium homeostasis is tightly regulated, and Mg competes with other ions, such as potassium and calcium, for transport channels in cell membranes, which are essential for maintaining intra and extracellular ionic balance and cellular functions.^58,72^ Alterations in Mg concentrations induced by omeprazole and other PPIs have been associated with cardiovascular disorders, requiring further investigation.^72–74^

To understand why this occurred, it is necessary to consider some factors related to magnesium metabolism, the interaction between minerals in the body and the impact of omeprazole on these interactions.^58,63^

Omeprazole may disrupt magnesium metabolism by affecting both the absorption and excretion. A reduction in gastric acidity interferes with intestinal Mg absorption, leading to compensatory mechanisms that influence its distribution in peripheral organs, including the spleen.^60,62,74^ Furthermore, because magnesium shares transport pathways with K and Ca, omeprazole-induced alterations in ionic balance may result in changes in Mg compartmentalization between intra and extracellular spaces.^59^

A possible explanation for the observed increase in spleen Mg is a compensatory redistribution in response to changes in ionic metabolism. If omeprazole interferes with K and Ca absorption or excretion, the organism may redistribute Mg in an unusual way to restore homeostasis.^61,63,75^

Figure 5c shows the potassium concentrations in the rat spleen samples. The treated group exhibited higher values than the control group at all treatment periods, reaching 247.37 μg·g^–1^ at 60 days versus 92.81 μg·g^–1^ in the control group, suggesting that omeprazole increases the spleen K concentration. Similar to Mg, K is not stored in large amounts in the spleen but can accumulate during inflammatory processes involving the influx of immune cells.^58,59^

Omeprazole, by altering gastric acidity, may indirectly affect mineral and electrolyte homeostasis, including potassium. Prolonged inhibition of acid secretion can lead to changes in mineral absorption and electrolyte balance, influencing the potassium distribution among tissues. Potassium is essential for maintaining the membrane potential and for several cellular functions. Thus, any imbalance can trigger compensatory mechanisms that redistribute K to different organs, including the spleen.^76^

Figure 5d represents Zn concentrations in the spleen, revealing consistently higher values in the treated group throughout the experiment (175.76 μg·g^–1^ at 60 days versus 60.61 μg·g^–1^ in the control group). These findings suggest that omeprazole promotes zinc accumulation in the spleen. As the spleen produces and stores immune cells such as lymphocytes, and Zn is essential for immune function, the elevated zinc levels may indicate increased immune activity or inflammatory responses induced by omeprazole.^12,20,54^ The mobilization of K, as discussed previously, may also be related to these inflammatory conditions.

Figure 5e shows that iron concentrations in the spleen increased over the treatment period in both groups, with significantly higher values in the treated group (1493.62 μg·g^–1^ versus 678.89 μg·g^–1^ in the control group at 60 days). The spleen acts as a major reservoir of Fe, and excessive storage may reduce circulating Fe levels, potentially contributing to iron deficiency anemia. One-way ANOVA confirmed a statistically significant difference between treated and control groups (*p* <0.05), indicating that prolonged omeprazole exposure significantly alters spleen Fe content. Similar results were reported by Naveh et al. (1987), who observed increased spleen Fe concentrations in rats treated with antacids.^64^ These findings suggest that drugs reducing gastric acidity can alter Fe storage in tissues, with possible implications for systemic iron homeostasis and anemia.

Figure 5f shows calcium concentrations in spleen samples, which were higher in the treated group across all periods, reaching 2974.97 μg·g^–1^ at 30 days compared to 1967.5 μg·g^–1^ in controls. This result indicates that omeprazole may promote Ca accumulation in the spleen. Since only about 1% of the body Ca is stored in tissues, including the spleen, such accumulation could indicate reduced bone Ca utilization, potentially associated with osteoporosis in the treated rats. Furthermore, elevated intracellular Ca has been linked to cardiovascular complications, such as hypertension, although the mechanisms remain unclear.^67–69^ One hypothesis suggests that omeprazole reduces calcium and other mineral absorption, lowering serum concentrations while increasing tissue deposition, thereby disrupting mineral homeostasis and contributing to elevated blood pressure.^68^

## Conclusion

This study evaluated the effects of omeprazole administration on the absorption and bioavailability of essential nutrients, focusing on Fe, Ca, Mg, Zn, Cu, and K. Integrated analysis of hematological and biochemical profiles, along with the elemental composition of rat organs, revealed that omeprazole treatment significantly altered mineral homeostasis and relevant physiological parameters. Evidence of iron deficiency anemia was observed, characterized by reduced circulating Fe, decreased hemoglobin levels, lower red blood cell counts, and altered hematimetric indices. In addition, reductions in Cu levels may impair intestinal Fe reabsorption. Alterations in Ca, Mg, and K concentrations also indicate potential effects on bone metabolism and cardiovascular function.

Overall, these results support the hypothesis that prolonged omeprazole use may interfere with nutrient absorption and promote systemic imbalances. Further investigations with extended treatment periods are recommended, particularly considering the chronic use of proton pump inhibitors in clinical practice.

## Data Availability

All data are available in the text.
